# Carcimona of the Stomach

**Published:** 1898-10

**Authors:** 


Carcimona of the Stomach.—Dr. Rumpel has reported a case of cancer of the stomach remarkable for certain clinical peculiarities. The patient was a man, 48 years of age, who had ailed for nine months, although the only symptom to which his cachectic condition could be attributed was an extremely abundant expectoration of muco-purulent masses which were ejected principally in the morning after an effort of coughing. The manner in which this expectoration was produced led to the suspicion of a pulmonary lesion. Microscopical examination showed the presence of pavement-epithelium and pus-corpuscles in the expectorated matter. As, however, every symptom of catarrh of the larynx was lacking and nothing abnormal was found in the lungs, the case was thought to be one of severe catarrh of the œsophagus, as is sometimes observed in malignant disease of the œsophagus or cardiac orifice.

The patient succumbed to the progressive cachexia, and at the autopsy there was found a cancer of the upper curvature and posterior wall of the stomach. The disease extended thence to the œsophagus. The latter tube was not constricted, and this fact explains the ease with which, during life, sounds could be passed into the œsophagus.—*La Tribune Medicale.*

